# Third party disability of family members of adults with dysphagia

**DOI:** 10.4102/ajod.v12i0.1040

**Published:** 2023-01-27

**Authors:** Kim Coutts, Bibi Sayed

**Affiliations:** 1 Department of Speech Pathology, Faculty of Humanities, University of the Witwatersrand, Johannesburg, South Africa

**Keywords:** dysphagia, caregiver, third party disability, ICF, patient centred care

## Abstract

**Background:**

Third-party disability (TPD) has been studied in multiple patients including those with aphasia and hearing loss. Only one study has been done in relation to caregivers of adults with dysphagia. Third-party disability has been analysed using the International Classification of Function and Disability (ICF) framework. This study, therefore, used the ICF model to explore TPD of caregivers of adults with dysphagia for the context of Johannesburg in South Africa.

**Objectives:**

To describe how caregivers experience TPD when caring for adults with a dysphagia in Johannesburg.

**Methods:**

Data were collected from five primary adult caregivers, who were all family members, from government clinics in Johannesburg. This article reports the findings from the interviews that were analysed thematically using a top-down analysis approach.

**Results:**

Caregivers experienced challenges related to TPD mostly related to difficulties of being able to do activities of daily living for themselves, their household chores and attending social engagements. The use of body structure and function from the ICF model was not overtly applicable to the caregiver population. A new visual representation has been suggested to highlight the key themes to augment the social and psychological changes as seen on the ICF framework and demonstrated the specific interaction that these factors had on one another.

**Conclusion:**

Third-party disability is present in caregivers of patients with dysphagia. Healthcare workers need to be aware of the impact that this can have when preparing home management strategies. This newly devised representation can assist in creating a locally relevant patient-centred care approach but requires future input.

**Contribution:**

This article has provided greater insight into TPD in caregivers of adult patients with dysphagia in an urban African context. It has led to new information that can be used as an adjunct to the ICF model when understanding this phenomenon.

## Introduction

Dysphagia is a swallowing disorder that can result from a variety of pathologies in adults, including strokes, degenerative neurological conditions, head injuries, respiratory conditions as well as head and neck cancers, to name a few (Cichero 2006). If dysphagia remains undiagnosed or poorly managed, it can result in severe consequences for the person, including dehydration, malnutrition and aspiration pneumonia, all of which can result in death (Kertscher et al. 2014). In a setting such as South Africa where the majority of the population relies heavily on the under-resourced public healthcare setting, avoiding negative consequences is vital to ensure a reduced hospital stay (Coutt & Solomon 2020). This also implies that the management of dysphagia at home also needs to be managed adequately by the family.

There are currently no epidemiological data on the prevalence of dysphagia in South Africa. This is possibly because of dysphagia being heterogeneous in nature. Based on the South African quadruple burden of disease profile, non-communicable diseases leading to stroke as well as trauma-related injuries are significantly high in this context (Pillay-Van Wyk et al. 2016; WHO 2011). A recent study confirmed that stroke is one of the top 10 causes of death in South Africa with over 25 000 annual deaths reported (Ranganai & Matizirofa 2020). Another study in the province of Kwa-Zulu Natal indicated a high incidence of traumatic brain injury and the resources in place to manage these individuals are inadequate (Jerome et al. 2017). From these data, it can be assumed that the resultant dysphagia present in this population would be high.

Although the management of dysphagia requires a multidisciplinary team (MDT), the speech-language pathologist (SLP) is often the case manager. In South Africa, where there is a high patient-to-SLP ratio (Pillay et al. 2020), the SLP will manage these individuals at both an in- and out-patient level. People who have been successfully discharged from the hospital will require their families to manage their dysphagia at home with the assistance of the SLP and other members of the MDT. This has some important implications, as a study by Coutt and Solomon (2020) highlighted that caregivers of adults with dysphagia do experience third-party disability (TPD) when managing dysphagia at home.

Third-party disability is defined as an able-bodied caregiver experiencing a ‘disability’ as a result of caring for a significant other (Nund et al. 2014). Third-party disability has been researched in people with aphasia (Grawburg et al. 2013) as well as hearing loss (Scarinci et al. 2009), but there is little known about this phenomenon in relation to caregivers’ managing adults with dysphagia specifically. According to Scarinci et al. (2009), the majority of caregivers experienced difficulties with participation in certain activities both at home and socially. This was supported in the one study in this area by Nund et al. (2014) that looked at TPD in caregivers of those adults with dysphagia resulting from head and neck cancers. Another study by Grawburg et al. (2013) found that there were limited data on this matter, which impacted on the understanding of TPD of caregivers with adults with aphasia. A local study conducted by Coutts and Solomon (2020), assessing the use of modified diets by caregivers at home in the South African context, their findings revealed despite not looking at TPD specifically, that caregivers have participation, environmental as well as personal factors affected. This significantly impacts on their daily functioning such as their ability to work and earn a living. Third-party disability, when referring to those studies above, has also predominantly been researched in economically developed countries, which has limitations when understanding the impact of TPD on caregivers in a setting such as South Africa. This is important as the majority of South Africans live below the poverty line and require the use of public hospital facilities (Coovadia et al. 2009; StatsSA n.d.).

**FIGURE 1:**
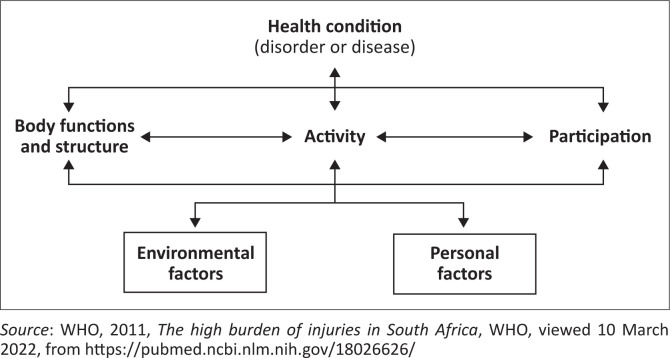
International classification of function and disability model.

The TPD studies mentioned above have utilised the International Classification of Function and Disability (ICF) in an attempt to understand this phenomenon. The ICF model is used to describe the function of a person with a disability in terms of body structure and function, activities of daily living and participation. It also includes both environmental and personal factors, which can be seen in Figure 1.

Therefore, this study chose to use the ICF model as a means for collecting and analysing the data. The aim of this study was, therefore, to describe how family members experience TPD when caring for adults with a dysphagia in Johannesburg.

## Research method and design

This was a descriptive qualitative study using phenomenological principles as the study aimed to describe the experiences of family caregivers looking after other family members with dysphagia. Once consent was obtained from each abovementioned party, full ethical clearance was granted and data collection could commence. Sites were various government clinics around Johannesburg. After ethical approval from the University of the Witwatersrand, data collection commenced. The researchers ensured that all ethical principles such as confidentiality and anonymity were adhered. There was also a distress protocol put in place.

The researcher used purposive sampling to obtain the first participant; thereafter, convenience sampling was used to recruit further four adult caregiver participants. The researcher contacted the clinics in the area and made the SLPs aware of the study. The clinic SLPs then informed potential participants who then voluntarily contacted the researcher if they showed interest to participate. Participants were included if they were over 18 years and needed to be the primary (main) caregiver. Participants needed to be proficient in English verbally as well as to be able to read and write in English because of the nature of the data collection tools. This was asked by the researcher prior to starting the data collection. The authors acknowledge that the inclusion criteria may have limited the type of data obtained from caregivers based on the inclusion criteria. This is important to consider when doing future studies on this topic. This was stated in the information letter that was sent out to the participants prior to them consenting for the study. The researcher also checked this prior to starting data collection with each participant. They were given an information sheet prior to the study, and if they chose to partake, they were given a consent form. There were three data sources for triangulation purposes. The same participants were used for all the three sets of data collection and followed the same recruitment strategy. These were, namely, the Adult Carer-Quality of Life (AC-QoL), the semi-structured interviews and the reflexive journal. The participants first answered questions from the AC-QoL questionnaire (Elwick et al. 2010). The results from the AC-QoL were used to inform the drafting of the interview questions. The questions that had the most weighting, that is the most significant, were included in the interview. This was determined by the questions that received the highest values from the participants. The aspects from the AC-QoL that were predominantly used in the interviews were support for caring, caring choice, caring stress, money matters and ability to care. Following this, individual semi-structured interviews were conducted by the researcher. The research was approved by the ethics committee for the use of an online platform to conduct the interviews in order to accommodate the coronavirus disease 2019 (COVID-19) lockdown-level restrictions at the time of data collection. Only one platform was used, which was Zoom. The audio from the interviews was recorded and then later transcribed by a research assistant. The interviews were conducted in English.

A pilot study was conducted with Participant 1 to ensure that the process of the data collection was in alignment with the aim of the study and that the data collection tools were correct. Participant 1 was a 49-year-old man who is the husband of a person with dysphagia. He is the full-time caregiver. The dysphagia and other behavioural complications of the individual were because of a head injury. A limitation was that because of the small number of recruited participants, the pilot study could only be conducted on one caregiver. No changes to the questions for the interview were deemed necessary as the researcher was able to obtain adequate data. The length of the interview was deemed appropriate, and the participant was able to respond to each question appropriately, so no rephrasing was needed. The results from the pilot study were used in the main data analysis. For the larger study, the researcher also used a reflexive journal during the data collection process for debriefing purposes and to ensure that her bias was not imposed on the data analysis as part of the rigour process. A research assistant, who was trained by the researcher, was used in the transcriptions to ensure that there was no confirmation bias and that the transcriptions were captured verbatim. The researcher and research assistant checked the transcription for accuracy and confirmability. Data were then analysed thematically using a top-down approach based on the aspects from the AC-QoL questionnaire and the ICF model that were used in the interviews and subsequent data analysis. The methodology as set out by Braun and Clarke (2006) was used for the data analysis as well as those on analysing data for a phenomenological approach in health care, which aims to describe a phenomenon, and in the case of this study, it was to describe the experiences of caregivers (Priest 2002). Table 1 describes the participants who participated in this study.

**TABLE 1:** Description of participants.

Participant number	Caregiver relationship	Age	Employment status	Cause of dysphagia	Length of being a caregiver	Time spent with adult with dysphagia
1 (pilot study)	Husband	49	Employed	Head injury	3 months	Entire day
2	Wife	75	Pensioner	Motor neuron disease	2 months	Entire day
3	Grandson	21	Unemployed	CVA	10 months	Entire day
4	Mother	47	Unemployed	Epilepsy	2 months	Entire day
5	Sister	40	Employed	CVA	3 months	6 h per day

CVA, cerebrovascular accident.

### Ethical considerations

This study was approved by the University of the Witwatersrand’s Human Research Ethics Committee (HREC). The ethics application was submitted to the university’s ethics committee, which allocated the researchers’ provisional acceptance to conduct the study pending site approval. This provisional ethics letter was then sent to each site together with the proposal and consent forms. Permission to conduct the study was requested from the Department of Health as well as the managers and Head of the Speech Pathology Departments at each site.

## Results

All participants were caregivers to people with both oral and pharyngeal phases of dysphagia that resulted in dietary modifications at home. Table 2 describes the diet modifications that were in place at the time of the interviews.

**TABLE 2:** Diet modifications that were being used at home.

Diet modification in place	*n*
Percutaneous endoscopic gastrostomy (PEG)	1
Pureé diet with thin liquids	2
Soft diet with thickened liquids	3

These diet modifications were important to describe as it also contextualises the results. This shows that all family members had to adjust the diet for the adult with dysphagia, which would be separate from the rest of the family. This would require time and other resources.

The results from the interview were analysed using the aspects of the ICF framework.

### The International Classification of Function and Disability model and third-party disability

Table 3 depicts how TPD impacted the caregivers according to the themes set out by the ICF framework. The ICF is the framework that has been used to understand and describe TPD and was, therefore, initially used in this study to analyse the data. The themes not relating directly to the ICF framework will be discussed separately.

**TABLE 3:** Findings from participants relating to the International Classification of Function and Disability model (*N* = 5).

ICF factor	*n*	Aspects affected
Body structure	1	Weight loss
Body function	4	Sleeping pattern disturbances
Activities and participation	5	Communication with the person is difficult because of impairment
		Caregiver having time to eat meals is reduced because of lack of time
		Time to do household shopping is reduced because of lack of time
		Household chores are reduced because of lack of time and financial pressure of not being able to buy supplies
		Social interaction is reduced because of increase in the number of responsibilities, lack of time and concern for society’s perception of persons’ behaviour (from diagnosis, e.g. temper)
		Meal preparation is difficult for diet modifications
		Ability to adequately perform their job and studies in reduced
		Time for leisure activities was reduced
Contextual and environmental factors	3	Financial difficulties because of difficulties to attend job and increased pressure of taking care of the individual
		Emotional concerns such as stress, anxiety and depression
		Use of coping strategies
		Education and counselling from MDT
		Family support
Independent factors	3	Access to transport COVID-19 effects on access Living space requirements

ICF, International Classification of Function and Disability; MDT, multidisciplinary team; COVID-19, coronavirus disease 2019.

It is evident that all of the participants experienced difficulties with the ICF aspects. The most predominant theme is the impact on activities and participation of the caregivers as well as environmental factors.

### The International Classification of Function and Disability factors

#### Body structure

Only one participant experienced weight loss, which was stress induced. During the interview, he stated ‘*Too much, I even lost weight*’ (Participant 3). This appears to be a novel finding and a significant one that will be discussed below. Despite it only being one participant, these findings need to be considered on a larger scale as weight loss does have further health implications.

#### Body function

Three out of the five caregivers reported that sleep patterns were interrupted because of the person requiring increased attention both day and night. This resulted in significant fatigue. In the interview, Participant 1 reported that he would be required to wake up every 2 h to monitor the individual but would find it difficult as seen in the quote, ‘*So at night, it even become more stressful because that’s the time when you lose track of time and you don’t watch the patient as you’re supposed to*’ (Participant 1). Another participant stated, in the interview, about a cause for the change in his sleeping pattern ‘*Yes, often because I am worried about her*’ (Participant 3). Participant 5 reported that she was unable to get adequate sleep as seen by ‘*I wasn’t sleeping enough*’ (Participant 5). This lack of sleep can have other negative health consequences for the caregivers. This lack of sleep is also interlinked with personal factors, such as the emotional state of the caregiver, which will be explored below. This is not directly linked to their dysphagia management and the constant risk of aspiration but being a caregiver in general.

Emotional factors were seen to be experienced in three out of the five participants in this study. The emotions that were experienced by family members included stress, frustration, worry, concern, depression and fear. Participant 1 described the experience of caring for the individual as ‘*a roller coaster of emotions*’ (Participant 1). For this participant, he noted how often he feels stressed, and this was related to the generalised care, which included feeding and other requirements such as maintaining personal hygiene for the person, providing medication, monitoring her temperature, percutaneous endoscopic gastrostomy (PEG) tube and tracheostomy care. Because of this, the theme was included in the interview for further discussion and analysis. He further supported this with the statement ‘… *you constantly stressed out because you constantly caring for that person*’ (Participant 1). Participant 3 was different and related specifically to providing care while eating as in the interview he stated, ‘*I don’t feel safe around her because anything can happen when she is eating …*’ (Participant 3). This was in relation to the high risk of aspiration as seen by ‘… *because the food doesn’t go through her throat, it goes through her vocal cords*’ (Participant 3). In the interview, Participant 5 reported that she would constantly worry about her sister during the night. She stated ‘*I had restless nights because here I am worried about her, her wellbeing*’ (Participant 5). This worrying is related to her eating and fear of aspiration and general care. This concern was also experienced when their loved ones either did not want to eat or take medication, as seen in the following quotes. ‘*I not worry too much, only when he not eat and when he sick*’ (Participant 4); ‘*No, it concerned me if he didn’t eat*’ (Participant 2) and ‘*A lot of the time, when she didn’t wanna take her pill*s’ (Participant 5). These emotional stresses of the caregivers are linked to other aspects such as poor sleeping patterns as well as limiting their social activities and participation in other activities. However, Participant 3 reported that their emotional state did get better with time as seen by the quote ‘*Only at the beginning, I felt very frustrated but then I got better used to her*’ (Participant 3). This is perhaps important to note for the MDT when counselling caregivers.

Although caregivers did experience concerns under *body function*, it is not predominant and often interlinked with other factors, such as psychological and emotional, which the ICF does not directly nor specifically account for.

#### Activities and participation

**FIGURE 2:**
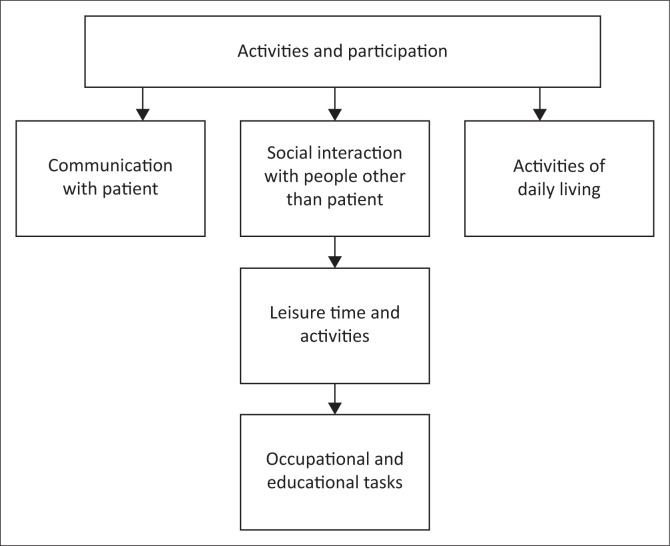
Sub-themes identified under activities and participation.

The following aspects of activity and participation were reported in the participants according to the ICF model, as shown in Figure 2.

It is evident from the diagram that the sub-themes do affect each other, which is often not reported on in the ICF framework. Each subtheme will be discussed individually, and the possible linkages will be explained.

#### Communication

There are two separate aspects related to communication that emerged from this study. The first is how communication is affected in relation to interactions with family members as well as socially in terms of reflecting on their challenges as a caregiver. This was because of two factors, namely gender and religion. Participant 1 stated ‘*As a male and also coming from a Muslim perspective, you tend to keep these things to yourself. You don’t ask for help …*’ (Participant 1). This difficulty with health-seeking behaviours of caregivers is a new finding in terms of its impact of TPD and needs to be addressed by all members of the healthcare team, especially when taking person-centred care into account (Santana et al. 2018). It is evident that personal factors, such as gender and religion, can clearly have an impact on other aspects, which have not been addressed in current models. This requires attention.

The other impacting factor on communication was the ability of the loved one to communicate effectively with the caregiver. This creates frustration as seen in the quote from Participant 3 ‘*I feel like I am stupid because I am explaining the same things over again*’ (Participant 3). This is not a novel finding but is something that treating healthcare professionals need to counsel caregivers about and be aware of in their management plan.

#### Activities of daily living

Two out of five participants reported experiencing difficulties in this area. Caregivers would complete their basic tasks, such as personal hygiene and eating, and then, the rest of the day was used to assist their loved one. Participant 1 stated, ‘*You constantly caring for that person, so you neglect your personal issues, even your personal hygiene*’ (Participant 1) and ‘*The most important thing you end up neglecting is eating. You stop eating or if you do, you eat very little*’ (Participant 1). Participant 3 echoed these concerns by stating ‘… *coz everything I need to do for me, I need to make it snappy because you can’t leave her for too long*’ (Participant 3).

Three out of the five participants reported that their ability to do grocery shopping was impacted upon. Often, they needed to reply on other people to assist them as stated by Participant 1 ‘… *but you know in terms of going to the shopping centre, I would just hand that to someone else*’ (Participant 1) and Participant 3, ‘*Yes coz I can’t even go for fifteen minutes, even to go to the tuckshop because you need to buy fast*’ (Participant 3). Participant 5 also reported a sense of urgency when shopping as she stated, ‘*I will go maybe just in and out you know getting something and back*’ (Participant 5).

In terms of chores, three of the five participants reported having difficulties with this. Participant 1 was unable to afford a domestic worker, as they were now a full-time caregiver as seen in the quote ‘*Basically got a patient in the room that is basically from a financial expenses is draining. You know you don’t have a stay in maid*’ (Participant 1). Participant 3 reported an inability to clean the house, ‘*I can’t even clean the house because I need to watch her consistent and see if she’s choking or not*’ (Participant 3). Whereas Participant 5 was only able to do what was necessary, ‘*The only house chores I cared about was to make up my bed and cleaning the small space that I am in*’ (Participant 5). These findings are important as this will have repercussions on the quality of life (QoL) of the caregivers and could result in more emotional distress because of an increase in caregiver burden (Namasivayam-MacDonald & Shune 2018). The impact of caregivers having an inability to participate in basic household functions links significantly to other emotional factors, such as stress and anxiety. This can then impact on their ability for them to see themselves as a ‘good’ caregiver.

Three of the five participants mentioned that meal preparation was affected for them. Participant 1 indicated that he struggled with maintaining PEG feeds in the beginning, but this improved with time. He also stated that when the COVID-19 pandemic started, people were less inclined to provide his family with support for general caring and assistance, which included meal preparation ‘… *there was a day where it was plentiful and there was a day where there was basically nothing*’ (Participant 1), which made the task of being a caregiver much harder. Because of having to prepare a modified diet, this increased the costs for meal preparation, ‘*We don’t usually buy the groceries that we used to buy you see. Some other things are added on the list and the money increased you see*’ (Participant 1). The impact of COVID-19 will also be addressed later as a separate factor.

Participant 3 indicated that he was required to prepare two meals, which were challenging as seen in the statement, ‘… *coz when I’m cooking, I have to make two meals. One for me and separate for her*’ (Participant 3). Whereas Participant 5 did not have difficulty with preparing two meals but rather it was a time factor for her ‘*It really didn’t matter like I was just cooking because people were hungry, they needed to eat*’ (Participant 5) and

‘We had a blender, we would cook vegetables for example, and afterwards we would just blend them and add a bit of water to become softer for us to feed her.’ (Participant 3)

The aspects of time and assistance need to be considered when implementing home programs for adults with dysphagia and their families.

#### Leisure activities

Four of the five participants indicated that their social interactions had been impacted upon. Participant 1 avoided social gatherings, as he wanted to avoid people who were seen as ‘*sympathising*’ (Participant 1) and felt obligated to assist him. He described his social life as ‘*Out the window*’ (Participant 1). Participant 3 never went anywhere as they felt that they were unable to leave their loved one at home unattended. ‘*Ha friends, I don’t go there anymore coz yoh it’s going to be difficult to leave her at home*’ (Participant 3). Participant 5 did not want to socialise anymore, ‘*I just wanted to be in my corner on my own just to deal with this. Like it affected my social life more than anything*’ (Participant 5).

Three of the five participants were unable to partake in leisure activities like they did before. This was supported in a statement made by Participants 1 and 3, ‘*It affected it 100%. There was absolutely no leisure, no pleasure. We were all just sitting out of concern*’ (Participant 1). ‘*I can’t even do fun activities anymore. I need to be with her 24/7*’ (Participant 3). In the interview, Participant 5 indicated that partaking in leisure activities ‘*was a luxury for me*’ (Participant 5). These are significant findings that can hinder the optimal functioning of the caregiver and relate to other factors such as emotional distress.

Participant 4 was a bit different in that they did not socialise because they felt that people in social settings did not understand the behaviours and feeding methods of their loved one ‘*Sometimes I don’t go anywhere because I know people don’t like how he acts or don’t understand*’ (Participant 4).

All of these different reasons for not wanting to socialise need to be acknowledged and addressed by team members when implementing person-centred care. This can also have an impact on the emotional status of the caregiver.

#### Occupational and educational responsibilities

Three of the five participants reported that they were unable to fulfil their occupational or educational responsibilities since being a full-time caregiver. Participant 1 stated ‘*I wasn’t doing 80% of my job …*’ (Participant 1), which indicates how negatively his job was impacted by. Participant 3 was unemployed but was unable to attend job interviews because of caregiver responsibilities. He also wanted to attend school again but mentioned ‘*Again, I want to go to school, I can’t*’ (Participant 3). These participants also indicated that they were unable to fulfil their jobs and educational tasks, which impacted on them negatively.

For Participant 5 who had a job and was studying, in the interview, she stated, ‘*If I go to work and do what I needed to do and then I’m out*’ (Participant 5), meaning that she was always required to be on leave. She also stated that she was failing certain aspects of her course. These are significant factors that caregivers need to be counselled on.

#### Environmental and personal factors

**FIGURE 3:**
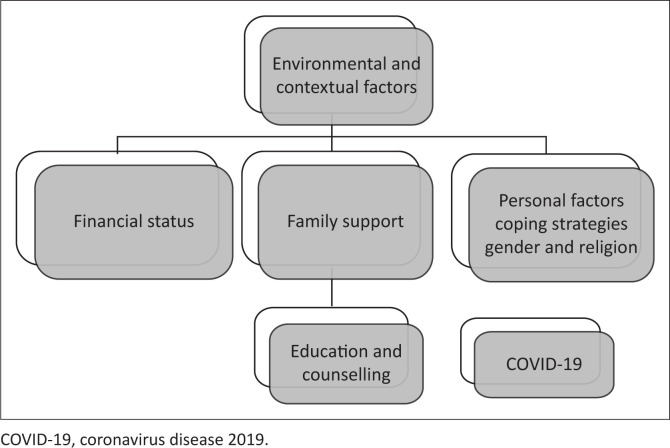
Sub-themes under environmental and contextual factors.

There were six themes that emerged under environmental and personal factors during the data analysis. This can be seen in Figure 3, and each theme will be discussed individually.

#### Finances

This was a significant theme as three of the five participants reported having had their financial status affected. Participant 1 had medical aid, but was burdened when healthcare professional costs were not covered by this service, he stated, ‘*It drained me totally*’ (Participant 1). He described it as ‘*It left me actually in a deficit but it was required*’ (Participant 1).

Participant 3 was unemployed as he described his situation as ‘*It costs us a lot, we don’t work and we have to look after her*’ (Participant 3), and the same feelings shared by Participant 5, ‘*Yes, yes, yes it did*’ (Participant 5), affect them financially as these adults were on a modified diet. This theme links to that of emotional stress and also impacts on the caregivers’ QoL. These circumstances need to be considered by all members of the healthcare team when addressing family needs for at-home management.

#### Family support and roles

Participant 5 was the only participant to indicate the shift in family roles since becoming a caregiver. He said ‘*You now need to play mother and father*’ (Participant 5). These personal adjustments can be difficult. Three of the five participants always felt that their life was on hold since becoming a caregiver, because they felt a sense of responsibility in needing to take care of them:

‘So everything that I did, I was mindful of my time all the time. Because I need to be there, I need to look after her, I need to see how she is doing.’ (Participant 5)

and ‘*So I couldn’t leave because I was feeling like I am abandoning her somehow*’ (Participant 5). Some of the caregivers felt that this situation was unexpected and ‘*forced*’ on them:

‘The reason why, this type of thing was basically forced upon me. Between the hospital and medical aid. They insisted that the patient be home for palliative care so it was forced on me.’ (Participant 1)

Sometimes, this left caregivers with a feeling of dissatisfaction.

Despite the hard changes and negative impacts being a caregiver has had on the participants, three of the five caregivers reported experiencing positive aspects related to caregiving. Findings revealed that some of the caregivers felt that their relationships with the loved ones were strengthened through this experience, especially as eating is a social and often family-centred event. This is a novel and interesting finding. Again, this needs to be accounted for in the counselling process and highlights that there are some positive elements in being a caregiver.

#### Personal factors

**Coping strategies:** The need for coping strategies was noted among three of the five participants. Participant 1 reported that praying was used as a strategy, because ‘… *the worst period of my entire life over the past one and a half years*’ (Participant 1). Participant 2 stated that by keeping her family member happy, helped her cope ‘*One has to be very cheerful and don’t show that they are a burden*’ (Participant 2). This was a similar strategy used by Participants 3 and 5 as they would hold hands and listen to music with their loved one to keep them happy, ‘*But every time I would hold her hand she would hold it back and squeeze it. That gave me courage and strength to want to see her healed*’ (Participant 5). By loved ones being happy, caregivers also felt more encouraged.

**Education and counselling:** Importantly, for healthcare workers, three of the five participants stated that receiving counselling from the healthcare team and having a better understanding of the medical diagnosis were beneficial:

‘For me, it was very difficult in the beginning. But fortunately, before taking her home, you had a nutritionist telling you this is what you need to buy, you know, lentils, you need to buy these types of foods, this, that and the other.’ (Participant 1)

Participant 3 suggested, ‘… *maybe taking me to classes to understand the condition and how it works, to understand some other things coz when she’s coughing, I don’t know what to do*’ (Participant 3). The carryover and counselling that need to be provided by healthcare workers are, therefore, of significance.

### Independent factors

#### Coronavirus disease 2019-related factors

One participant reported on the impact that the level 5 lockdown had on his ability to take care of his loved one as he was able to work from home:

‘I was just fortunate enough, like I said, this happened during lockdown, I was working from home and I could, I could, you know play between my work and watch over her.’ (Participant 1)

but that social gatherings were less because of the lockdown restrictions. ‘*I basically stayed away from social gatherings. But also, again, the whole episode was in lockdown*’ (Participant 1). This resulted in loneliness as well as perhaps their ability to seek help from others when preparing meals and for the feeding of the person. These are the experiences from a few caregivers, but the impact of COVID-19 on the functioning of families still needs to be explored, because this may have longer-term impacts despite lockdown restrictions mostly being lifted.

## Discussion

The findings from this study reveal that caregivers of adults with dysphagia experience TPD predominantly related to difficulties of being able to do activities of daily living for themselves, their household chores and attending social engagements. This study highlighted the stress of being a caregiver and how this hinders their ability to perform other daily activities. The current study highlighted that the emotional and psychological well-being of caregivers is a central theme and thus most other factors branch off of that. This is central for understanding and describing TPD. This correlates with the other studies done in TPD such as those by Nund et al. (2016), Scarinci et al. (2009) and Authors (in press), which related back to the themes of the ICF, mainly around activities and participation as well but also including environmental and personal factors.

Within that, in a context such as South Africa, there are multiple complex contextual, environmental as well as cultural factors to consider, as shown in Table 3. These factors include aspects such as family dynamics, gender roles as well as family support and religion. The details from this study are novel and need to be explored further.

During the data analysis phase as described in the results, the authors found that the ICF model underplays certain themes that are relevant for caregivers. The framework does not always highlight the influence that each aspect can have on each other, and it also shows that each factor is represented equally, whereas this study showed that certain factors should be more heavily weighted than others when it comes to TPD. The factors highlighted in this study need to be further explored and described in greater detail than what the ICF framework currently offers. These aspects need to be explored further in future research on a larger sample size and in different contexts. The ICF model could be used as a generic model to understand TPD, but as this is a complex phenomenon with multiple factors influencing it, a more specific representation to depict this as a possible adjunct to the ICF model is required. Caregivers’ needs are different from the person who they are caring for, and thus, a different model to the ICF is required to fully describe their specific experiences. In order to address the aim of the study of describing experiences of TPD of caregivers, the authors felt that a potential new visual representation was needed, which stemmed from the results of this study.

**FIGURE 4:**
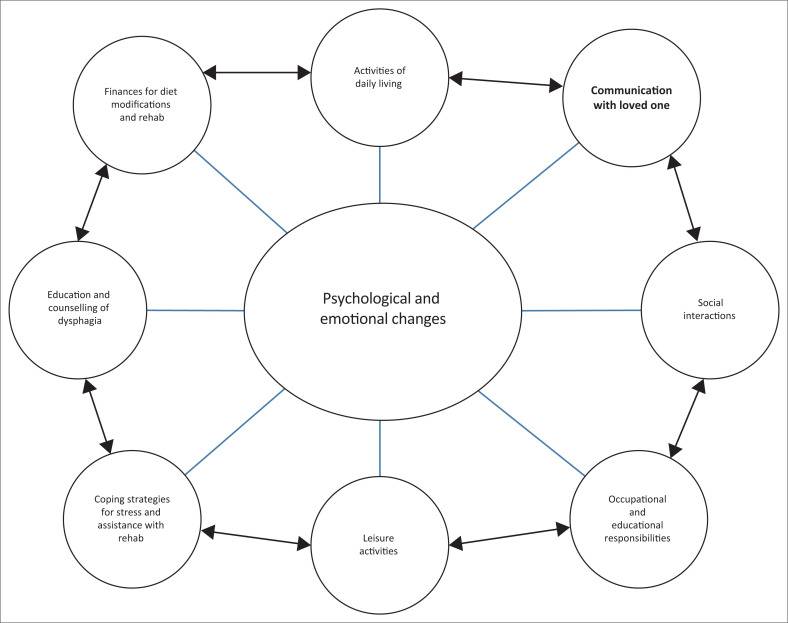
Suggested visual representation for understanding the impact of third-party disability of family members for adults with dysphagia in an economically developing context.

The themes that arose are multifactorial and impact on one another creating complexity. As a result, this study has suggested a potential visual representation in order to demonstrate how TPD presented in this study. This suggested that the visual representation of TPD has also attempted to highlight the connections that these themes have on one another. This representation could be used as part of the ICF to augment certain factors or could be used independently. This representation is based on the descriptions of the participant’s experiences. As these findings were based on a small sample size, this representation does require further research to enhance and develop it; however, it is a start in understanding TPD in caregivers of adults with dysphagia in Johannesburg. This visual representation can be seen in Figure 4.

This new visual representation will assist healthcare workers in understanding the impact of TPD on family members in Johannesburg. This is important when devising home programs for adults with dysphagia. Healthcare workers would need to consider all of the aspects in the figure when considering management strategies for the home environment. It will also be important healthcare workers when counselling caregivers on what to expect upon discharge and when they are settling into a home routine. This is visual framework that has provided new information from which healthcare workers can draw for the above purposes. This can also assist in the referral process to ensure that caregivers receive the necessary support.

South Africa is a diverse country with multiple factors that can influence the health-seeking behaviours of this population and those factors need to be accounted for (Otwombe et al. 2015). These factors are seen in the different coping strategies used by the caregivers and how different cultural and gender factors influenced caregivers’ ability to seek help and ultimately affect their QoL as seen in one or two participants in this study (Reinhard et al. 2008).

Byun et al. (2016) stated that caregivers experienced higher levels of stress and anxiety. As seen by the results of this study, as reflected in Figure 4, these emotions can be linked to various factors, including sleeping patterns, finance worries, difficulty completing work responsibilities as well as the inability to attend social and leisure activities. In terms of avoiding social activities, this is not uncommon in caregivers as seen by other studies (such as Howells et al. 2021; Nund et al. 2016; Patterson et al. 2013). However, it is important to note the impact that this lack of social interaction can have on the QoL of the caregiver. This ultimately will increase the other emotional concerns again. If caregivers are also avoiding activities of leisure, this can negatively impact on their ability to promote their own sense of well-being (Oliveira, Sousa & Orrell 2019).

Not being able to fulfil work responsibilities can also have negative consequences on the financial status of the family. This is important as adults with dysphagia often require modified diets, which can come with an increase in costs. This was seen in the study by Authors (in press). It is of significance for team members to consider the impact of finances when developing a home management plan for people (Gardiner et al. 2020). The impact that COVID-19 had on South African families, in terms of work and finances, needs to be further explored in relation to TPD as well as the impact of lockdown still has long-lasting effects, especially in terms of employment and finances.

However, the importance of caregivers incorporating coping strategies and having adequate counselling from the healthcare team prior to discharge was seen as a positive factor for these caregivers. It is the role of the rehabilitation team to consider person-centred care to ensure effective outcomes and support with the families (Reinhard et al. 2008). This aspect is, therefore, incorporated into the visual representation.

The limitation of this study is that of the sample size was small, but the findings of this study have provided more detail on TPD for caregivers of adults with dysphagia. However, this new visual representation will allow for future studies on TPD to be more tailored to the specific needs of the caregivers, so that this phenomenon can be better understood. This visual representation does require more input and refinement from more research as this was based on a small sample size in a specific setting. The researchers have, therefore, stipulated that this visual representation needs to be piloted on multiple caregivers in different contexts.

As this study stemmed from a larger one, the standardised measure of the AC-QoL was used. This was to assist in the methodology and interpretation of the findings from the participants. It can, therefore, be recommended that future studies use this visual representation and possibly a different methodology to further explore these findings.

This study was also done with participants who were in the more acute phase of caregiving. The findings from this study also need to be explored in caregivers who have been doing this for a longer time.

## Conclusion

Third-party disability is a common occurrence in caregivers of adults with dysphagia. This study has shown that TPD has a general negative impact on the QoL of these caregivers. The main aspect affected is that around psychological and emotional changes that they experience as a result of doing home caring and management of the disability. This management is related to their general medical condition as well as the dysphagia. Caregivers find that there is little time to do daily tasks including work and educational responsibilities. These can lead to financial burdens as rehabilitation strategies can be expensive. However, the importance of counselling by the healthcare team and providing caregivers with education is paramount. Previous studies that used the ICF framework in an attempt to understand TPD were unable to fully explore all aspects of TPD as the ICF framework presents with short comings when focusing on the caregiver specifically. This study, therefore, proposed a newly developed visual representation of TPD that can be piloted in other studies relating to TPD, which includes novel themes and the potential impact that these themes have on each other. Future studies need to include caregiver experiences of those in different contexts such as rural areas and those who are not fluent in English and who are not proficient in reading and writing.
